# Comparison of predictive effect of the dietary inflammatory index and empirically derived food-based dietary inflammatory index on the menopause-specific quality of life and its complications

**DOI:** 10.1186/s12905-023-02485-y

**Published:** 2023-07-01

**Authors:** Niloufar Haghshenas, Fatemeh Hosseini Baharanchi, Ebru Melekoglu, Mohammad Hassan Sohouli, Farzad Shidfar

**Affiliations:** 1 Department of Nutrition, School of Public Health, Iran University of Medical Sciences, Tehran, Iran; 2 Department of Biostatistics, School of Public Health, Iran University of Medical Sciences, Tehran, Iran; 3 Faculty of Health Sciences, Nutrition and Dietetics Department, Cukurova University, Adana, Turkey; 4 Student Research Committee, Department of Clinical Nutrition and Dietetics, Faculty of Nutrition and Food Technology , Shahid Beheshti University of Medical Sciences, Tehran, Iran

**Keywords:** Dietary Inflammatory Index, Empirically derived food-based dietary inflammatory index, Menopause-specific quality of life, MENQOL

## Abstract

**Introduction:**

Menopause, defined as the cessation of menstruation for at least 12 months, is one of the important stages of a woman's life cycle. Some hormonal variations occur during the transition to menopause, which affects women’s quality of life. Recently, the role of dietary factors in alleviating symptoms has been investigated.

**Aim of this study:**

We tried to investigate the relationship between dietary inflammatory index (DII), food-based dietary inflammatory index (FDII) and quality of life, and menopausal symptoms, comparing their predictive power and suggesting the best cut-off point.

**Methodology:**

One hundred forty-nine postmenopausal women were included in the cross-sectional study. After collecting data by interview, the desired variables were calculated. Logistic regression and ROC curves were used to investigate the relationship and predictive power of DII and FDII with menopausal symptoms.

**Findings:**

We observed that both DII and FDII were significantly associated with the severity of sexual symptoms. The first tertile of DII (OR = 0.252, *P*-value = 0.002) and FDII (OR = 0.316, *P*-value = 0.014) had a significantly lower odds ratio for severe to moderate symptoms compared to the third tertile. Both inflammatory indices had significant predictive power in predicting the probability of having severe to moderate poor quality of life (FDII (*p*-value = 0.004) > DII (*p*-value = 0.006)) and sexual symptoms (DII (*p*-value = 0.002) > FDII (*p*-value = 0.003)). Also, regarding the physical subtype, only FDII (*p*-value = 0.002) results were significant.

**Conclusion:**

Both dietary inflammatory indices appear to be suitable for predicting quality of life, but FDII had slightly more predictive power. It seems that the quality of life and severity of menopausal symptoms may be improved, particularly with regard to sexual symptoms, by following an anti-inflammatory diet.

## Introduction

Menopause, defined as the cessation of menstruation for at least 12 months [1], is one of the natural and important stages of every woman's biological life cycle [2]. It occurs on average around 51 years of age [3]. The most fundamental change in this period is the drastic decrease in endogenous estrogen and the consequent cessation of ovarian function [4].

Postmenopausal women make up one-third of the United States female population, and millions of them are in transition [5]. It is estimated that by 2050, approximately one-fifth of the world's female population will be at this stage [6]. In 2016, it was announced that approximately 8.5 million women aged 40–64 live in Iran [6]. We know that this stage accounts for more than 33% of most women's lives [7]. This period is associated with mental, genitourinary, and physical complications that reduce health-related quality of life (HRQOL) to varying degrees [8]. Women experience various symptoms such as mood instability, depression, night sweats, hot flashes, sexual disorders, vaginal dryness, sleep disturbance, and anxiety [9, 10]. The transition to menopause is not abrupt and usually takes about seven years (or even up to 14 years) until a woman reaches menopause [11]. Surprisingly, menopausal symptoms can begin ten years before menopause and last more than ten years afterwards. Hormonal changes, especially the drastic decrease in estrogen, are the main factors in the etiology of these complications [12]. Dietary modifications, such as reducing fat consumption, increasing fruit, whole grain, and vegetable consumption, and weight loss were effective in improving vasomotor symptoms [13]. Systemic inflammation has recently been recognized as an important factor in psychiatric disorders. A prospective study in Australia reported a 20% reduction in the risk of depression with an anti-inflammatory diet [14]. Also, it has been reported that the risk of anxiety, depressive disorders and psychosis is higher in women with the highest inflammatory food pattern score [15–18]. We can evaluate the inflammatory potential of the diet with two indices called DII and FDII. While DII is based on the amount of 45 selected nutrients [19], FDII uses specific food groups [20]. As mentioned, the complications of menopause affect different aspects of women's lives. On the other hand, the high population of this group can cause a great medical and economic burden on societies. Lifestyle and diet have shown a significant effect on the severity of these symptoms in various ways, such as modifying inflammation levels in recent studies. In this study, we aimed to investigate for the first time the relationship between DII and FDII and quality of life and menopausal symptom levels including vasomotor, psychological, physical and sexual symptoms. Finally, the power of DII and FDII to predict moderate to severe reviewed complications was compared and cut-off points were suggested with the highest accuracy.

## Materials and methods

### Study participants

This research procedure lasted approximately one year, from June 2021 to June 2022, and studied 149 postmenopausal women referred to healthcare centers in Lar, Iran. Women who met the inclusion criteria were selected for the interview and the participants consisted of women older than 40 who had not experienced a menstrual period for at least 12 months. Other inclusion criteria were: 1) Not suffering from mental illnesses, cancer, autoimmune disorders, and other serious illnesses. 2) In the presence of diabetes, high blood pressure, or thyroid disorders, the disease must be completely under control. 3) No chronic smoking 4) BMI between 18.5 and 40 kg/m^2^. Women with these characteristics were excluded: 1) consuming a diet with less than 800 kilocalories (kcal) or more than 4000 kcal 2) using hormone therapy or dietary supplements 3) taking psychiatric drugs 4) being unwilling to maintain cooperation. Our study protocol was approved by The Research Ethics Committee of the Iran University of Medical Sciences.

### Assessment of general characteristics, socioeconomic status, anthropometry, and physical activity

The standard general information questionnaire was used for sociodemographic, family, medications, dietary supplements, health conditions, and medical records. Weight (to the nearest 100 g) and height (to the nearest 0.5 cm) were measured with a Seka brand digital scale and wall-mounted tape measure according to standard instructions. BMI (kg/m^2^) was calculated for each individual based on the collected anthropometric measurements. We used the General Physical Activity Questionnaire (GPPAQ), an easy tool for adults (16–74 years old) [21], to assess the physical activity levels of the participants. This questionnaire consists of three questions about the individual's physical activity level at work, total physical activity duration in the last week, and walking speed. Finally, the participants were categorized into four physical activity levels. All of these data were collected by trained researchers through face-to-face interviews.

### Dietary assessment

Evaluation of participants' food intake in the past year was assessed with a 117-item food frequency questionnaire adjusted for the Iranian population, the validity and reliability of which have been confirmed in previous studies [22]. Consumption amounts for 117 items were assessed using standard and conventional serving sizes. Each person reported the frequency of consumption of each food item based on the 9 suggested answers (ranging from never or less than once a month to 6 times or more per day). The reported values for each item were converted to daily consumption amounts (grams) and each individual's energy, micronutrient and macronutrient intakes were obtained using USDA tables [23] and the Iranian-modified version of IV Nutritionist software.

### Inflammatory indices

#### DII

This score is calculated based on the FFQ data obtained. Full details of this index and its development have been reported elsewhere [19]. Briefly, 1,943 articles reporting the effect of dietary components on inflammation biomarkers (including C-reactive protein, IL-6, IL-1b, IL-4, IL-10, and TNF-a) were reviewed and anti-inflammatory components were scored as -1, ineffective diet factors as 0, and dietary components with pro-inflammatory properties as + 1. And eventually, 45 dietary factors including flavonoids, spices, macronutrients and micronutrients were extracted from this extensive literature search and used to calculate DII.

The mean and standard deviation of the inflammatory score and global intake for each of these 45 parameters are provided by the investigator. To calculate this score for each individual, the first step is to subtract the mean intake of each 45 intended dietary parameter from the corresponding global mean intake and then divide the results by the global standard deviation to obtain the Z score. In the next step, the Z score should be converted to percentile and then multiplied by 2 and minus one for reducing skewness. The numbers obtained for each item are multiplied by the corresponding inflammatory score. As a final step, scores for all 45 parameters are summed and the total dietary inflammatory score is calculated for each individual. DII can range from -8.87 to + 7.98 but has been reported between -5.5 and + 5.5 in most previous studies. The lower the score, the higher the dietary anti-inflammatory power, and the higher the score, the more inflammatory it is. We computed DII using 30 dietary factors, energy, protein, carbohydrate, total fat, saturated fatty acids, vitamin B12, iron, and cholesterol were pro-inflammatory items and thiamin, riboflavin, niacin, vitamin B6, folic acid, zinc, selenium, magnesium, garlic, onion, black tea, caffeine, n-3 and n-6 fatty acids, mono and polyunsaturated fatty acids, fiber, β-carotene, and vitamins A, C, D, and E were anti-inflammatory components. Other dietary factors of DII score were not used due to a lack of data. All dietary factors were adjusted for energy intake by the residual method before starting the calculation of this index [24]. Fortified food consumption was not reported by any participants.

#### FDII

Another index used to evaluate the dietary inflammatory profile is FDII. Tabung et al. [20] suggested that there should be another indicator combining nutrients, as nutrients are not consumed alone in the daily diet and synergistic effects of nutrients may limit the interpretability of this profile. Therefore, they established a new inflammatory index based on food groups (EDII) rather than nutrients to provide more comprehensive information from DII. In this study, we used FDII, the Iranian food-based version of EDII, first compiled by Salari-Moghadam et al. [24] based on data found in a study of female teachers in Tehran [25].

We computed the average daily intake of 28 special food groups (13 anti-inflammatory items including fruits, natural juices, fish, poultry, vegetables (cabbage family, green leafy, yellow, and other vegetables) tomatoes, beans, whole grains, low-fat dairy products, and tea and 15 inflammatory food groups including processed meats, red meat, eggs, high-fat dairy products, coffee, potatoes, fried potatoes, pizza, refined grains, mayonnaise, sweetened beverages, sweets and desserts, butter, hydrogenated fats, and vegetable oils). Same as DII, we initially adjusted all values for energy using the residual method. The numbers were then multiplied by the corresponding factor load extracted from the reference study. Finally, the scores of all food groups were summed and the result was divided by 100 to reduce the magnitude of the numbers.

### Assessment of menopausal symptoms

We used the menopausal-specific quality of life questionnaire (MENQOL) to assess menopausal symptoms and their severity. This questionnaire includes 29 items and four different subgroups of menopausal complications [26]. Of the 29 questions in this questionnaire, 3 assess vasomotor symptoms, 7 evaluate psychological, 16 physical, and 3 sexual problems. The validity and reliability of the Persian version of MENQOL were investigated by Mr. Falahzadeh [27] and his colleagues. For each question, the participant states whether she has experienced these symptoms in the past month. If the answer is yes, the participant gives a score between 0 and 6 according to the severity of the symptom experienced. Quality of life score is obtained by summing all these scores. A higher MENQOL total score indicates a lower quality of life.

### Statistical analysis

Mean (standard deviation) and frequency report (percentage) were used to describe quantitative and qualitative variables. The normality of all variables was tested with graphical methods and Kolmogorov–Smirnov (or Shapiro Wilk) test and logarithm (log) transformations were performed for non-normal variables. For performing analysis, DII and FDII scores were initially categorized as tertiles. A one-way analysis of variance and chi-square tests were used to compare demographic information and food intakes between 3 different groups of each model. Multiple regression analysis was performed on the role of demographic variables in MENQOL and the reported scores of its subgroups were evaluated. Three different tests were performed to investigate the relationship between these two inflammation indices and menopausal symptoms. We compared the mean (standard deviation) of the overall score of the questionnaire and the tertile groups of DII and FDII by analysis of variance. Pearson's correlation test was used to quantitatively assess the relationship between dietary indices and scores. In the final stage, we divided each subgroup into 2 levels. For this purpose, all scores were summed by two and ranged from 2 to 8. Not experiencing symptoms was also considered as 1 point. The average score of each subclass was calculated and if it was above 5, it would be classified as "moderate to severe", otherwise it was considered "mild or no symptoms". Eventually, logistic regression was used to compare the odds ratio for each of the symptoms to have severe to moderate intensity in DII and FDII tertiles for both raw and adjusted data. Age (years), length of time since menopause (years), energy (Kcal), job (employed or not), education (academic or not), physical activity, underlying diseases (having controlled diabetes, high blood pressure or thyroid disorder or not), and body mass index (kg/m^2^) were adjusted as confounding variables. All components of DII and FDII, including food groups, micro and macronutrients, and energy intake were interned in the regression model to assess their role in the resulting scores for each subgroup. Finally, to evaluate and compare the predictive power of DII and FDII regarding each of the symptoms examined, the ROC curve was plotted and a cut-off point was determined for each pattern with the highest sensitivity, specificity, and accuracy.

## Results

**Fig. 1:**
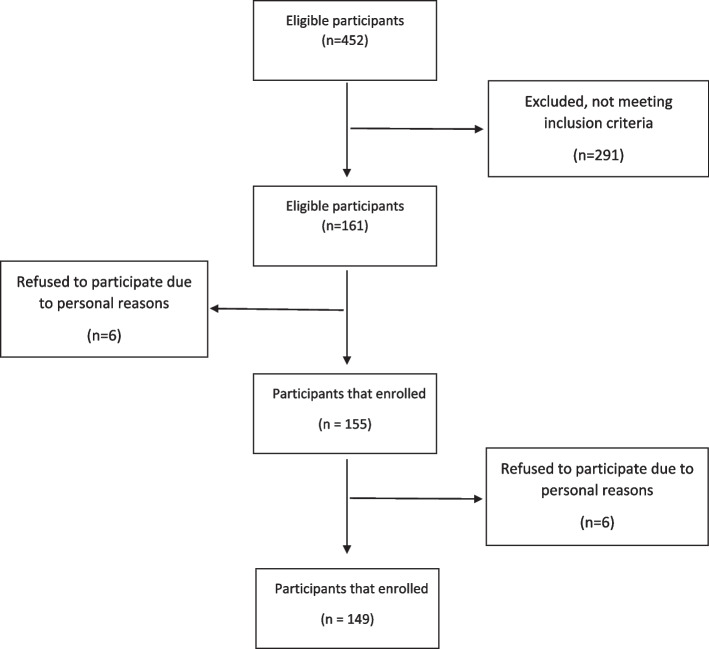
Flow diagram of participant enrollment

A total of 149 postmenopausal women aged 42 to 63 years were examined in this study. Figure 1 shows the participants’ enrollment. The distribution of the general characteristics of the participants by DII and FDII tertiles is shown in Table 1 and 2. There was no association between the DII score and general characteristics. Regarding FDII, only occupation (*p*-value = 0.001) had a significant *p*-value. The results of comparing the average food intake across the tertiles of indices are shown in Tables 3 and 4 (due to the large size of the tables, we only reported the significant items). Participants in the 3^rd^ tertile of DII (who had the diet with the lowest anti-inflammatory properties) had a higher intake of energy, total fat, and MUFA and a lower intake of vitamins B (B9, B6, B3, B2, B1), A, and C, β-carotene, fiber, protein, iron, and magnesium. In the 3^rd^ tertile of FDII, average intakes of soft drinks, fried potatoes, salt, and refined grains were higher and vegetables, fruits, tomatoes, tea, and nuts were significantly lower in the third tertile of FDII (all *p*-values < 0.05).

**Table 1:** Comparison of quantitative demographic variables in DII and FDII tertiles^1^

		**DII**							**FDII**						
		**T1** -3.29- _ 4.49		**T2** -2.66- _ 3.28		**T3** -0.99- _ 2.65			**T1** -6.46—_ 11.23		**T2** 4.78- _ 6.41		**T3** 1.16- _ 4.76		
**Variable**		Frequency	Frequency percentage	Frequency	**F**requency percentage	Frequency	Frequency percentage	*p*-value^2^	Frequency	Frequency percentage	Frequency	Frequency percentage	Frequency	Frequency percentage	*p*-value^2^
**Marriage status**	Married	41	27.50	42	28.20	41	27.50	0.85	44	29.5	40	26.85	40	26.85	0.52
	Other	9	6.10	7	4.70	9	6.10		6	4.00	10	6.70	10	6.10	
**Education**	Diploma or less	40	26.85	37	24.85	40	26.85	0.82	44	29.50	34	22.9	39	26.15	0.050
	Academic	10	6.70	12	8.05	10	6.70		6	4.00	16	10.75	10	6.70	
**Husbands’ education**	Diploma or less	38	25.50	40	26.80	37	24.85	0.64	38	25.50	36	24.15	41	27.50	0.373
	Academic	12	8.05	9	6.10	13	8.70		12	8.05	14	9.40	8	5.40	
**Occupation**	House keeper	40	26.85	38	25.50	36	24.15	0.62	47	31.54	32	21.48	35	23.49	0.001
	Other	10	6.70	11	7.40	14	9.40		3	2.00	18	12.10	14	9.40	
**Husbands’ occupation**	Self-employment	22	14.70	15	10.05	20	13.40	0.37	19	12.75	19	12.75	19	12.75	0.527
	Retired	16	10.75	23	15.40	13	8.70		22	14.70	15	10.05	15	10.05	
	Other	12	8.05	11	7.40	27	18.10		9	6.10	16	10.75	15	10.05	
**Underlying disease**	Yes	25	16.80	24	16.10	23	15.40	0.91	25	16.80	28	18.80	24	16.10	0.75
	No	25	16.80	25	16.80	27	18.10		25	16.80	22	14.70	25	16.80	
**Menopause type**	Hysterectomy	6	4.00	3	2.00	4	2.70	0.78	4	2.70	5	3.40	4	2.70	0.903
	Natural	44	29.50	46	30.90	46	30.90		46	30.90	45	30.20	45	30.20	
**Physical activity**	Inactive	9	6.10	12	8.05	15	10.05	0.08	13	8.70	8	5.40	15	10.05	0.22
	Relatively inactive	6	4.00	5	3.40	10	6.70		6	4.00	5	3.40	10	6.70	
	Relatively active	8	5.40	14	9.30	4	2.70		8	5.40	9	6.10	9	6.10	
	Active	27	18.10	18	12.10	21	14.10		23	15.40	28	18.80	15	10.05	

^1^ANOVA test was used for quantitative variables

^2^ *p*-value below 0.05 is considered significant

**Table 2:** Comparison of qualitative demographic variables in DII and FDII tertiles^1^

	**DII**				**FDII**			
	**T1** -3.29 _ -4.49	**T2** -2.66 _ -3.28	**T3** -0.99 _ -2.65		**T1** -6.46_ -11.23	**T2** 4.78 _ -6.41	**T3** 1.16 _ -4.76	
**Variable**	**Standard deviation ± mean**	**Standard deviation ± mean**	**Standard deviation ± mean**	***p*** **-value**^**2**^	**Standard deviation ± mean**	**Standard deviation ± mean**	**Standard deviation ± mean**	***p*** **-value**^**2**^
**Age, year**	3.29 ± 55.23	3.4 0 ± 54.55	2.95 ± 54.75	0.559	3.29 ± 55.23	3.40 ± 54.55	3.29 ± 55.23	0.278
**BMI, kg/m**^**2**^	4.12 ± 27.25	4.52 ± 27.83	4.45 ± 27.85	0.739	4.12 ± 27.25	4.52 ± 27.83	4.12 ± 27.25	0.434
**Time since menopause, years**	4.16 ± 5.44	4.28 ± 4.79	4.49 ± 5.50	0.669	4.16 ± 5.44	4.28 ± 4.79	4.16 ± 5.44	0.783
**Gravida**	2.05 ± 3.44	1.50 ± 3.02	1.89 ± 2.60	0.076	2.05 ± 3.44	1.50 ± 3.02	2.05 ± 3.44	0.204

^1^Chi-square test was used to compare the averages of qualitative variables

^2^ *p*-value below 0.05 is considered significant

**Table 3:** Comparison of average consumption of nutrients in DII tertiles^1^

**Dietary factors**	**T3**	**T2**	**T1**	
	Standard deviation ± mean	Standard deviation ± mean	Standard deviation ± mean	*p*-value^2^
Energy, kcal/d	469.18 ± 2240.09	410.22 ± 2045.13	411.28 ± 2027.96	0.026
Vitamin B9، mg/d	72.01 ± 495.01	65.73 ± 526.90	63.80 ± 576.45	< 0.001
Vitamin B6 ، mg/d	0.20 ± 1.61	0.19 ± 1.72	0.19 ± 1.83	< 0.001
Vitamin B2، mg/d	0.26 ± 1.42	0.31 ± 1.52	0.27 ± 1.57	0.037
Vitamin B3، mg/d	2.70 ± 18.91	2.53 ± 20.42	1.92 ± 20.90	< 0.001
Vitamin B1، mg/d	0.31 ± 1.61	0.26 ± 1.69	0.19 ± 1.76	0.022
B-caroten، mg/d	988.93 ± 2853.02	1166.83 ± 3844.07	1636.70 ± 4885.08	< 0.001
Fat, g/d	14.41 ± 71.99	11.62 ± 70.52	9.19 ± 65.63	0.023
Fiber ، g/d	12.27 ± 49.45	13.04 ± 50.99	11.77 ± 57.13	0.006
Iron ، mg/d	1.74 ± 14.82	1.59 ± 15.68	1.56 ± 16.70	< 0.001
Magnesium، mg/d	65.05 ± 315.33	59.60 ± 338.54	47.21 ± 385.12	0.001
MUFA, g/d	6.41 ± 26	6.35 ± 25.47	4.41 ± 22.62	0.009
Protein، g/d	6.94 ± 59.78	7.73 ± 64.82	6.85 ± 66.17	< 0.001
vitamin A، RE	106.83 ± 349.15	131.94 ± 457.56	157.14 ± 555.57	< 0.001
vitamin C، mg/d	50.66 ± 174.10	52.04 ± 182.01	62.98 ± 232.43	< 0.001

^1^ANOVA test was used to compare the averages

^2^ *p*-value below 0.05 is considered significant

**Table 4:** Comparison of average consumption of nutrients in FDII tertiles^1^

**Dietary factors**	**T3**	**T2**	**T1**	
	Standard deviation ± mean	Standard deviation ± mean	Standard deviation ± mean	*p*-value^2^
Tea، g/d	202.87 ± 280.20	212.45 ± 334.21	309.78 ± 493.73	<0.001
Fruit, g/d	113.40 ± 436.19	118.45 ± 558.70	165.10 ± 675.29	<0.001
Yellow vegetables, g/d	16.89 ± 18.14	7.57 ± 11.37	25.29 ± 21.15	0.026
Tomato, g/d	45.27 ± 101.65	50.91 ± 122.81	54.01 ± 144.14	<0.001
Other vegetables, g/d	48.17 ± 166.96	44.67 ± 181.07	61.50 ± 211.06	<0.001
Fried potatoes, g/d	12.45 ± 5.94	4.45 ± 2.02	3.11 ± 1.61	0.011
Refined grains, g/d	118.92 ± 378.38	83.87 ± 290	95.02 ± 249.86	<0.001
Nuts, g/d	8.87 ± 7.93	9.02 ± 11.28	19.51 ± 14.86	0.039
Drinks ،g/d	68.19 ± 50.09	18.28 ± 10.25	34.75 ± 15.93	<0.001
Salt, g/d	0.65 ± 4.31	0.65 ± 3.93	0.61 ± 3.94	0.004

^1^ANOVA test was used to compare the averages

^2^ *p*-value below 0.05 is considered significant

When the relationship between demographic variables and MENQOL scores was examined, only the duration of menopause had an inversely significant relationship with the general and psychological scores at the 5% significance level, controlling for the effect of other factors level. The physical score was found to be inversely related to age and directly related to the duration of menopause. Regarding sexual scores, only BMI and physical activity had significant *p*-values. None of the investigated variables showed a significant association with the vasomotor subgroup (data not reported).

### Association of DII and FDII with MENQOL

As we mentioned above, the association between the scores of DII and FDII and menopausal symptoms were analyzed with three different tests. None of the correlation coefficients from Pearson's correlation test was significant (data not reported). Results for comparing the mean scores of MENQOL and its subgroups in DII and FDII tertiles were also not significant in the raw or adjusted models (data not reported). In the final step, the odds ratio of suffering from severe to moderate levels of each symptom across tertiles of dietary indices were compared through logistic regression and the following results were obtained: DII (*p*-value = 0.002) and FDII (*p*-value = 0.014) had a significant inverse relationship with the odds ratio of having severe to moderate level of menopausal sexual symptoms. However, the other relationship investigated were not statistically significant (Table 5). Analyses of the effects of food groups and nutrients showed that higher intakes of vitamin C and low-fat dairy products improved quality of life, and greater amounts of snacks, iron, butter, and juices increased overall questionnaire scores. Dietary intakes could predict 58% of MENQOL changes in this model. None of the dietary items had a statistically significant association with vasomotor symptoms, but higher amounts of low-fat and high-fat dairy products, niacin, and vegetables were associated with less psychological domain severity. Higher intakes of vitamin C, folate, and low-fat dairy products were associated with less physical symptom severity, however, it showed a positive relationship with consumption amounts of vitamin A, iron, juices and snacks. Finally, the amount of potato consumption of the participants was inversely related to sexual symptoms, while the amount of fruit juice consumption and iron intake were directly related to the severity of menopausal complications. Moreover, these results showed that this model could predict 59% of changes in scores for psychological, sexual, and physical symptoms (data not reported).

**Table 5:** Multiple odds-ratio for menopausal complications among DII and FDII tertiles^1^

	DII					FDII				
	**T1**	**T2**		**T3**		**T1**	**T2**		T3	
	OR	OR (95% CI)	*p*-value^2^	OR (95% CI)^3^	*p*-value^2^	OR	OR (95% CI)	*p*-value^2^	OR (95% CI)	*p*-value^2^
**MENQOL**										
Crude	1.00	0 (–0)	0.997	0 (–0)	0.997	1.00	0 (–0)	0.997	0 (–0)	0.997
Adjusted^4^	1.00	0 (–0)	0.997	0 (–0)	0.997	1.00	0 (–0)	0.997	0 (–0)	0.997
**Vasomotor symptoms**										
Crude	1.00	0.474 (0.171–1.315)	0.152	0.803 (0.320–2.015)	0.640	1.00	0.587 (0.217–1.593)	0.296	0.870 (0.342–2.212)	0.769
Adjusted	1.00	0.380 (0.126–1.144)	0.085	0.818 (0.305–2.190)	0.689	1.00	0.687 (0.236–2.006)	0.493	0.939 (0.327–2.696)	0.907
**Psychological symptoms**										
Crude	1.00	0.546 (0.149–1.999)	0.361	0.392 (0.095–1.613)	0.194	1.00	1.152 (0.405–3.277)	0.790	0.446 (0.125–1.590)	0.213
Adjusted	1.00	0.451 (0.114–1.782)	0.256	0.451 (0.114–1.782)	0.262	1.00	0.214 (0.039–1.180)	0.077	0.361 (0.086–1.521)	0.165
**Physical symptoms**										
Crude	1.00	0.489 (0.085–2.803)	0.422	0.479 (0.084–2.743)	0.409	1.00	0.122 (0.014–1.036)	0.054	0 (–0)	0.997
Adjusted	1.00	0.473 (0.071–3.136)	0.438	0.713 (0.105–4.850)	0.729	1.00	0.098 (0.009–1.044)	0.054	0 (–0)	0.997
**Sexual symptom**										
Crude	1.00	0.261 (0.112–0.610)	0.002	0.254 (0.109–0.593)	0.002	1.00	0.540 (0.242–1.207)	0.133	0.303 (0.129–0.714)	0.006
Adjusted	1.00	0.253 (0.105–0.607)	0.002	0.252 (0.104–0.609)	0.002	1.00	0.598 (0.256–1.397)	0.235	0.316 (0.126–0.791)	0.014

^1^The results have been obtained using logistic regression

^2^A significance level of less than 0.05 is considered

^3^Odds ratio (95% confidence interval)

^4^Adjusted for age, time since menopause, energy, occupation, education, marriage, physical activity, underlying disease, BMI

### Comparison of the predictive power of indices

Both DII or FDII had significant predictive power for general symptoms of menopause and quality of life (MENQOL) based on their ROC curves. The area under the curve shows the predictive power of each index (Table 6), and about MENQOL, the power of FDII is more than the other one (DII < FDII). Scores of -4.3137 and -2.44 for FDII and DII, with 77.81% and 81.9% accuracy, can be suggested as a suitable cut-off point for estimating the possibility of suffering from severe to moderate general menopausal symptoms. In fact, the probability of encountering someone with a score equal to or less than -4.3137 for FDII and -2.44 or less for DII is 77.81% or 81.9%, respectively. Regarding vasomotor and psychological groups, neither DII nor FDII had significant predictive power. However, the physical subgroup showed significant predictive power (*p*-value = 0.002, the area under the graph = 0.822, best cut-off point = -4.704) for FDII. Similar to the overall score, both indices had a statistically significant power to predict moderate to severe menopausal sexual symptoms. However, DII (0.652) had a slightly larger area under the curve than FDII (0.647) and the suggested cut-off points are -5.8920 for FDII and -3.015 for DII.

**Table 6:** Comparison of the predictive power of the menopausal symptoms by DII and FDII

Menopausal symptoms	Dietary index	Area under the curve	Confidence interval	*p*-value^1^	Suggested cut off point	Accuracy	Specify	Sensitivity
**MENQOL**	FDII	0.926	(0.831–1)	0.004	-4.3137	77.81%	77.2%	100%
	DII	0.905	(0.836–0.974)	0.006	-2.44	81.9%	81.4%	100%
**Vasomotor**	FDII	0.547	(0433–0.661)	0.424				
	DII	0.519	(0.400–0.638)	0.743				
**Psychological**	FDII	0.607	(0.438–0.777)	0.187				
	DII	0.605	(0.450–0.760)	0.198				
**Physical**	FDII	0.822	(0.695–0.949)	0.002	-4.704	72.9%	72%	87.5%
	DII	0.642	(0.423–0.861)	0.176				
**Sexual**	FDII	0.647	(0.554–0.740)	0.003	-5.8920	61.1%	56.4%	69.1%
	DII	0.652	(0.557–0.747)	0.002	-3.015	62.5%	58.5%	69.1%

^1^A significance level of less than 0.05 is considered

## Discussion

Our results indicated that both inflammatory indices have significant predictive power for severe to moderate general and sexual complications of menopause. Regarding the physical subgroup, only the effect of FDII was statistically significant, And the results regarding vasomotor and psychological symptoms were not. Furthermore, after assessing the relationships between these inflammatory indices and MENQOL reports, we observed a direct significant association between DII and FDII scores and the odds ratio of having strong to moderate sexual symptoms. As our research is the first to evaluate and compare the relationship and predictive power of two inflammatory indices with menopausal symptoms, the results cannot be compared with other articles. However, some articles examine the effect of certain dietary patterns on menopause-related problems. For example, some recent articles observed that greater adherence to the Mediterranean diet is associated with a reduced likelihood of death from any cause and cardiovascular-related deaths [28–30]. George and his colleagues [31] evaluated and compared different dietary patterns related to death in postmenopausal women. In this cohort study, the Mediterranean diet was observed to be associated with a reduced risk of death from cancer, death from any cause, and CVD. Generally, following a healthier diet, has been associated with a lower risk of death from chronic diseases. High consumption of plant-based foods, vegetable oils (especially olive oil) and fish oil, moderate consumption of red wine, and low consumption of animal-based foods are key features of the Mediterranean diet and have much in common with the anti-inflammatory diet model we examined in our study.

Recently, it has been declared that hormonal fluctuations in the menopausal process affect microbiota composition in all body parts. We also know that various diseases and complications such as osteoporosis, diabetes, autoimmune diseases, weight gain, fat deposition, breast cancer, inflammation and oxidative stress of the body, and mental problems are affected by our microbiota [32]. And this fact may explain the increased risk of such disorders in postmenopausal women [31, 33]. It has been declared that the changing vaginal microbiota during the transition to menopause is effective in experiencing sexual complications related to menopause. All these issues have a great impact on the quality of life of postmenopausal women [34]. On the other hand, some dietary indices, including EDII and DII, were identified as a predictor of the metabolism and composition of the microbiota, as well as the level of oxidative stress and inflammation in the body [35]. Therefore, we can conclude that following a diet with a higher anti-inflammatory score may predict better health status and higher quality of life for postmenopausal women. In the study of Soleimani et al. [36], three dietary patterns were defined among menopausal women i) vegetables and fruits (VF) that are inversely associated with general and mental symptoms; ii) mayonnaise, liquid oil, sweets, and desserts (MLSD) that have a direct significant relationship with general and genital symptoms; iii) solid fat and snacks (SFS), which are directly related to general and mental symptoms. The VF pattern has a lot in common with the anti-inflammatory diet and the other two patterns have pre-inflammatory properties. We also observed that higher consumption of fatty and sweet snacks, butter, and juices (similar to the SFS pattern) was associated with higher general and physical symptoms. Dairy products, especially low-fat, had a significant inverse association with general, physical and psychological symptoms. There was an inverse relationship between vegetable consumption and psychological dysfunction, and between vitamin C intake and general and physical subgroups. Therefore, it can be said that the results of this study have similarities and differences with our research. It is worth mentioning that in Soleimani’s study, menopausal symptoms were evaluated through the MRS questionnaire and the number of participants was higher than ours. A cross-sectional study conducted in Spain in 2020 found no significant association between adherence to a Mediterranean diet and different menopausal symptoms [37]. An interventional study of 17,473 postmenopausal American women studied the effect of dietary (reducing dietary fat and increasing fruits, vegetables, and whole grains) and weight changes on the severity of vasomotor symptoms. It was reported that both weight loss and dietary adjustments can be effective in relieving the symptoms experienced independently [13] Dietary adjustments in this study increased the anti-inflammatory score of the individual's diet. Therefore, we can say that the results of this study are consistent with our results. The inverse relationship between weight loss and hot flashes was also confirmed by another study [38]. It’s worth noting that the average body mass index of our study participants was also in the overweight range, perhaps we could see stronger significant effects if weight loss is considered. Another study on women in the transitional phase of menopause showed that the severity of vasomotor and physical symptoms in vegetarians was significantly lower than in omnivores [39]. The authors stated that physical and vasomotor symptoms peak during the menopausal transition period and tend to decrease thereafter, So, the results seem reasonable. This may also explain the lack of association observed for these subgroups in our study.

In addition, similar to our study, eating more sweets was associated with greater severity of physical symptoms. Another study, after analyzing data from 3302 women aged 42–52 years before full menopause, observed that no dietary factor is associated with the severity of vasomotor symptoms [40]. In a study evaluating 726 Chinese women with high blood pressure, whole plant foods pattern (whole grains, vegetables, and fruits) was associated with less severe symptoms [41]. This pattern exclusively had a specific inverse relationship with physical symptoms and nervous tension, and the association with vasomotor symptoms was not statistically significant, like our results. In our results, consuming more vegetables had a significant inverse relationship with the psychological group, and an inverse but insignificant relationship with general and physical scores. Safabakhsh et al. [42] showed an inverse association between vegetable and fruit consumption and overall MRS and somatic score in their study of 393 participants. In a study by Abshirini et al. [43], there was a significant inverse relationship between dietary total antioxidant capacity (DTAC) and total MRS, psychological and somatic scores. Another study in Iran by Aslani et al. [36] concluded that DII has a direct relationship with the somatic subgroup score of the TMRSS questionnaire. In our study, only the relationship between DII and sexual symptoms was significant. It should be noted that there are important differences between these two studies. While the DII scores in Aslani's data ranged from the inflammatory to anti-inflammatory zone (-4.33 to + 4.21), in our study, DII scores were only in the anti-inflammatory zone (-0.99 to -4.49) with varying intensities. Also, DII was composed of 34 food factors in Aslani's study, but 29 factors were in our study due to the different food questionnaires used. Menopause questionnaires, data analysis method and the number of participants are other differences between these two studies.

Studies are limited and contradictory, especially on urogenital symptoms. In this study, both DII and FDII had a clear inverse association with such complications. In fact, adherence to a diet with a higher anti-inflammatory power reduces severe to moderate sexual disorders in menopausal women. In a study by Soleimani et al. [44], a diet with high amounts of sweets, oils and, desserts was associated with increased severity of these symptoms. On the contrary, DII [36] and DATC [43] hadn’t any significant relationship with the symptoms analyzed. Surprisingly, the consumption of vegetables and citrus fruits was directly related to this subgroup in Safabakhsh et al.’s study [42]. In our results, higher amounts of iron and fruit juice consumption had a direct significant association with sexual scores, but potato consumption had an inverse relationship with this score. There was also a direct relationship between fruit and vegetable consumption with increased intensity of sexual symptoms, but it was not statistically significant. Safabakhsh stated that the carbonate found in vegetables may lead to alkaline states in the urinary tract and more vaginal atrophic symptoms in postmenopausal women [42, 45, 46]. Moreover, fruits and juices with acidic properties may act as possible bladder stimulants. However, nurses' health studies did not find any association between acidic fruit and the progression of urinary incontinence [42, 47]. In Bauckman's study, a dietary iron restriction was associated with lower urinary tract infections in rats [48]. Unfortunately, other similar previous studies did not report the relationship between sexual symptoms and diet.

Body inflammation levels can affect female sexual function through different mechanisms. Cytokines have a strong effect on the central nervous system and specific areas related to sexual processes, such as the mesolimbic reward system, thalamus, and cingulate cortex [49, 50], directly [51, 52] and indirectly by interactions with neurotransmitters [53]. Animal studies have also shown the reducing effect of pro-inflammatory cytokines on sexual interest in female rats [54, 55]. A small study of premenopausal women also indicated that higher CRP and IL-6 levels were associated with lower levels of libido, arousal, and sexual pleasure [56]. In general, it seems that inflammation can impair sexual desire and arousal. Moreover, it can also increase sexual aversion as a kind of defensive response to reduce the chance of infection [57].

Inflammation can also affect the production and activation of estrogen and progesterone, reducing their stimulating effects on women’s sexual desire and arousal [57–59]. Chronic inflammation may lead to endothelial dysfunction and impair nitric-oxide-mediated vasodilation [60, 61]. This process is essential for genital arousal. We know that one of the signs of inflammation is pain. Therefore, another way that can reduce sexual performance and desire is the development of pain during intercourse [57, 62].

Various factors such as cultural, social and personal beliefs can affect an individual's sexual quality and performance. In one Chinese study, the question regarding vaginal dryness was omitted due to cultural incompatibility [41]. Therefore, this may be the reason why no firm conclusions have been reached in this field so far [36, 63], and extensive clinical trials need to be designed in the future.

There are some points that should be considered in our study. Our study includes a specific population, most of them from the same city and with relatively similar social and cultural characteristics. Furthermore, all calculated DII and FDII scores were less than zero, and all evaluated women followed an anti-inflammatory diet. If these scores were more diverse, analyses between tertiles would be more significant. In addition, we know that the quality of life in the postmenopausal period is related to different and various factors such as the woman’s genetics and race, emotional and physical health, socioeconomic status, beliefs, and experience of stressful life events [64, 65]. Therefore, it seems unrealistic to expect to see strong associations between all menopausal complications and dietary factors.

This is the first study to compare and evaluate the predictive power of two different dietary inflammatory indices and their relationship to menopausal quality of life, which is the main strength of our research. We tried to test different statistical methods to find the best results and to provide a broader view to the reader. Finally, we tried to set appropriate cut-off points to make the results more practical at the clinical level. Other strengths of this study were the high accuracy of the interviewer, the use of questionnaires suitable for the Iranian population, the consideration of numerous possible confounding factors, and the strict inclusion criteria. This study also has limitations that should be noted. This was a cross-sectional study, so we were not able to determine exact cause-effect relationships. We experienced some limitations in sampling due to the Covid-19 pandemic. Data collection was done using interviews and this method is based on the participant's memory, so some mistakes are inevitable. In addition, the participant may give unrealistic answers to especially sexual and psychological questions for cultural reasons. We have to keep in mind that the results of the current study cannot be generalized to the average Iranian postmenopausal women living in other regions of the country and to other populations of different cultures and ethnicities.

## Conclusion

In conclusion, our results indicated that both dietary inflammatory indicators (FDII, DII) can be used to predict the rate of severe to moderate menopausal complications. DII was only able to significantly predict the overall score and sexual symptom levels, but FDII also had significant predictive power for the physical subgroup. The predictive power of FDII for MENQOL was slightly greater than that of DII, with the suggested cut-off point of -4.3137. These results need to be confirmed by clinical studies to provide a comprehensive guideline for improving the quality of life of postmenopausal women, taking into account other social and cultural conditions.

## Data Availability

Data is available upon request from the corresponding author for the article due to privacy / ethical restrictions.
